# Combined arthroscopic release with corticosteroid hydrodilatation versus corticosteroid hydrodilatation only in treating freezing-phase primary frozen shoulder: a randomized clinical trial

**DOI:** 10.1186/s12891-022-06065-3

**Published:** 2022-12-17

**Authors:** Zhu Dai, Quanhui Liu, Bo Liu, Ke Long, Ying Liao, Biao Wu, Wen Huang, Chao Liu

**Affiliations:** Department of Orthopaedics, the First Affiliated Hospital, Hengyang Medical School, University of South China, Hengyang, 421001 Hunan China

**Keywords:** Frozen shoulder, Corticosteroid injection, Hydrodilatation, Arthroscopic release

## Abstract

**Background:**

There has been no ideal treatment for freezing-phase frozen shoulder to rapidly relieve pain and improve joint mobility. No any other team directly compared the effectiveness of combination of arthroscopic release and corticosteroid hydrodilatation with corticosteroid hydrodilatation only in treatment of freezing-phase frozen shoulder.

**Methods:**

Seventy-two patients with freezing-phase frozen shoulder were randomly assigned to combined arthroscopic release with corticosteroid hydrodilatation (group A) or corticosteroid hydrodilatation only (group B). Clinical states were examined at baseline and periodically (Weeks 1, 4, 12, 24 and 1 year) after intervention by passive ROM; visual analog scale (VAS); UCLA and Disabilities of the Arm, Shoulder, and Hand (DASH) score.

**Results:**

The passive ROM, VAS, UCLA and DASH scores always improved along the time points (all *p* < 0.01). The passive abduction (pAB), passive forward flexion (pFL), passive external rotation (pER), passive internal rotation (pIR) were better in group A than in group B at Week 1, 4, 12, 24 (all *p* < 0.01). At 1 year post-operation, the pFL and pIR were better in group A than in group B (all *p* < 0.01). VAS scores of group A were similar with those of group B (all *p* > 0.01), the differences between group A and group B were all lower than minimal clinically important difference (MCID). At Week 12, the UCLA sores and DASH scores were 26.8 ± 3.8, 14.2 ± 2.0 in group A versus 22.3 ± 3.4, 22.5 ± 3.1 in group B (all *p* < 0.01). At Week 24 post-operation, there were 32.7 ± 2.0, 9.8 ± 1.5 in group A versus 26.3 ± 3.6, 17.5 ± 3.5 in group B (all *p* < 0.01). At 1 year post-operation, there were 34.5 ± 0.8, 1.7 ± 1.5 in group A versus 32.1 ± 2.3, 8.8 ± 2.8 in group B (all *p* < 0.01), the differences of UCLA scores between group A and group B at Week 24 was higher than the MCID. There were no complications such as infection, nerve or vascular injury.

**Conclusions:**

Combined arthroscopic release with corticosteroid hydrodilatation would yield better results in passive ROM and function than corticosteroid hydrodilatation only.

**Trial registration:**

ChiCTR1900024235, July 2, 2019 (Retrospectively registered).

**Supplementary Information:**

The online version contains supplementary material available at 10.1186/s12891-022-06065-3.

## Background

Frozen shoulder, also known as adhesive capsulitis, is primarily manifested as shoulder joint pain, with active and passive movement limitation. Its incidence in the general population is 2–5%, climbing to 20% in patients with diabetes [1]. This condition may be primary or secondary in nature. Typically, frozen shoulder is self-curative, with three overlapping phases: freezing phase (2–9 months), frozen phase (4–12 months), and thawing phase (5–26 months) [1]. In terms of pathophysiology, inflammation at onset of frozen shoulder leads to proliferation, thickening, and contraction of the synovial tissues [2, 3]. Treatment of frozen shoulder is aimed at pain relief and restoring shoulder ROM and function.

The freezing phase is marked by progressive shoulder pain and gradual decline in passive shoulder movement [1]. Patients at this point have the strongest desire for treatment, which is largely conservative [4] and includes oral non-steroidal anti-inflammatory drugs, physiotherapy, intra-articular corticosteroid injection [5–7], hydrodilatation or distention arthrography [4, 8], and various alternative local treatments (ie, fire needle in China) [9]. Some authors consider hydrodilatation with corticosteroids as the most effective conservative management for frozen shoulder, quickly relieving pain by reducing inflammation [4, 10]. However, a thickened and contracted joint capsule does not readily soften, so improving ROM is a relatively slow process [11]. Arthroscopic contracture release is an effective means of improving ROM and often reserved for frozen-phase or refractory frozen shoulder [12, 13]. As for patients with freezing-phase frozen shoulder, it does not effectively combat the severe ongoing inflammation, so recurrence risk is high [14]. Thus, there has been no ideal treatment for freezing-phase frozen shoulder to rapidly relieve pain and improve joint mobility.

There was no evidence on the effectiveness of combination of arthroscopic release and corticosteroid hydrodilatation in treatment of freezing-phase frozen shoulder, which has not been previously completed by any other team. Hence, the purpose of this study was to determine the effect of arthroscopic release combined with corticosteroid hydrodilatation in treatment of freezing-phase frozen shoulder.

## Methods

### Participants

Between August 2018 and January 2021, a total of 97 patients were treated for freezing-phase primary frozen shoulder by our senior surgeon. The following inclusion criteria were applied: patients with primary frozen shoulder, aged from 40–70 years old, with normal X-ray findings and global passive motion limitations as follows: < 100º forward flexion, < 10º external rotation, and internal rotation below L5 level. Freezing-phase frozen shoulder was stipulated as marked nocturnal pain (VAS > 7 [scored as 1–10]) of prolonged duration (≥ 2 months but < 9 months) [1], refractory to at least one course of physiotherapy. The following were grounds for exclusion: 1) shoulder stiffness secondary to previous trauma or surgery, 2) concomitant rotator cuff tear or subacromial impingement proven by MRI, 3) poor general health precluding surgery or corticosteroid injection, and 4) concomitant diabetes. Among the 97 patients, 10 were not in freezing phase and 5 had concomitant rotator cuff tears or subacromial impingement, 10 had diabetes, leaving 72 patients for random assignment. The protocol for the study was approved by the Institutional Review Board of The First Affiliated Hospital of University of South China. All participants provided informed consent before their participation in the study.

### Randomization

An independent physicians performed the recruitment and baseline examination at the outpatient department. After baseline examination, the enrollees received one of two sealed opaque envelopes marked as A or B, A would be assigned to combined arthroscopic release with corticosteroid hydrodilatation group (group A), B would be assigned to corticosteroid hydrodilatation only group (group B). 72 patients were assigned with the ratio of 1:1, with 36 patients in each group.

### Combined arthroscopic release with corticosteroid hydrodilatation

In group A, arthroscopic release and corticosteroid hydrodilatation were performed by the same group of surgeons, rotator interval and anterior glenohumeral joint (including superior, middle, and anterior part of inferior glenohumeral ligaments) was released, then subacromial interval was routinely checked, no debridement and decompression were needed. Full passive ROM was confirmed by manipulation. Injections (as described for group B) were done intraoperatively and at postoperative Weeks 1 and 4.

### Corticosteroid hydrodilatation only

In group B, triamcinolone acetonide (50 mg) and ropivacaine (100 mg) were mixed in saline to a volume of 20 ml. As an outpatient procedure, we injected 15 ml into glenohumeral joint posteriorly and 5 ml into subacromial space laterally, using anatomic landmarks. Patients were treated in sitting position, marking bodily puncture points and routinely sterilizing the skin. For injecting glenohumeral joint, a 6-cm needle entered 1 cm lateral to and 1 cm below the posterior angle of acromion, 30º relative to coronal plane. Injection was delivered upon sensing a breakthrough, confirmed by intraarticular fluid aspiration and ultrasound. For subacromial injection, a NO 5 needle introduced obliquely (~ 30º relative to horizontal surface) to the lateral surface of acromion was then moved inferiorly in increments until sensing a breakthrough, then injection was delivered and confirmed by ultrasound. Treatments were repeated 1 and 4 weeks after initial injection.

### Rehabilitation

Patients were educated to perform home exercise program and received regular follow-up at orthopedic clinic. In group A, daily rehabilitation was focused on forward flexion, external rotation, and deltoid muscle training, beginning 1 day after surgery to the extent tolerated. In group B, deltoid muscle training was engaged in a similar regimen, range of motion training was performed step by step to the extent tolerated.

### Follow-up assessments

After therapeutic intervention, passive ROM and VAS scores were assessed at Weeks 0 (baseline), 1, 4, 12, 24, and 1 year, obtaining UCLA scores and DASH scores at Weeks 0, 12, 24, and 1 year. The passive ROM including abduction, forward flexion, external rotation (arm at side), and internal rotation were primary outcome. PAB, pFL, and pER were measured using a goniometer. PIR was equated with the highest vertebral level of the back accessible by tip of thumb, described by Sun [7]. A clinical researcher blinded to group allocation was tasked with collection of patient data.

### Sample size calculation

Sample sizes were determined prior to randomization by test for two means with non-zero null utilizing MedSci sample size tools (version 2.1, Medsci, Shanghai, China). Based on our preliminary study of 15 patients, 36 participants in each group were required to detect a significant difference in pAB, with a power of 80% at a type I error level of 0.05, given an expected dropout rate of 20%.

### Statistical analysis

The analyses analyzed by per protocol principle. All measured data were expressed as mean ± standard deviation (SD) values. Intergroup comparisons were performed by *t*-test, using analysis of variance (ANOVA) for intragroup comparisons. Paired *t*-test was applied between time points, and data counts expressed as rates (%) were compared by chi-square test. Standard software (SPSS v21.0; IBM Corp, Armonk, NY, USA) facilitated above computations, setting significance at *p* < 0.01.

## Results

**Fig. 1:**
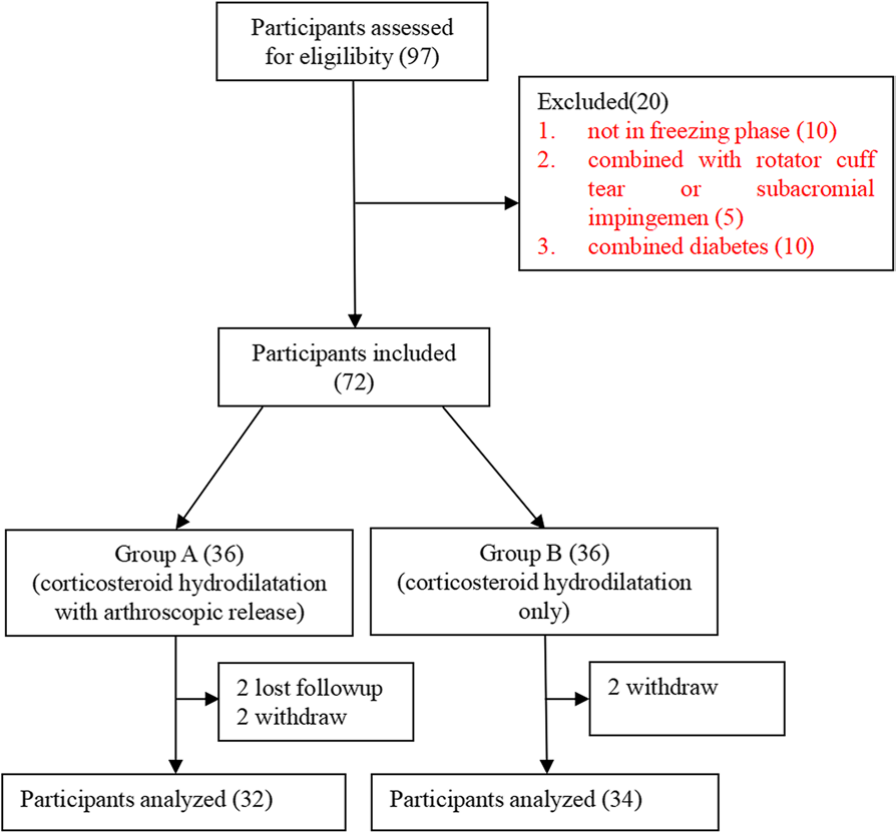
The flow chart of the patient through the study

During the one year monitoring period, all patients complied to the treatment and crossing over did not happen. some patients were lost to follow-up (group A, 2) or withdrew from the study (2, each group), leaving 66 patients (group A, 32; group B, 34) for analysis (Fig. 1). Demographics in each group were similar (Table 1), and the clinical characteristics of patients did not differ significantly at baseline (Week 0) (*p* > 0.01) (Table 2). There were no complications such as infection, nerve or vascular injury. The participants reported no adverse events during the treatment period, nor were any identified during the one year follow-up.

**Table 1:** Baseline characteristics of the participants

Characteristics	Group A (*n* = 32)	Group B (*n* = 34)
Age (y, mean ± SD)	53.3 ± 6.8	52.8 ± 7.1
Male vs Female	4 vs 28	5 vs 29
Height (cm, mean ± SD)	161.3 ± 4.7	162.6 ± 5.6
Weight (kg, mean ± SD)	58.2 ± 4.4	58.0 ± 5.9
Side (left vs right)	17 vs 15	19 vs 15
Duration (months, mean ± SD)	5.4 ± 2.3	4.5 ± 2.1
Smoking (yes vs no)	4 vs 28	5 vs 29
Cardiac disease (yes vs no)	6 vs 26	7 vs 27
Thyroid diease (yes vs no)	2 vs 30	2 vs 32

**Table 2:** Difference of outcomes at different time points between groups(x ± s)

Outcomes	0 week	1 week	4 weeks	12 weeks	24 weeks	1 year
pAB, deg						
Group A	69.6 ± 18.2	123.7 ± 24.9	144.0 ± 22.8	159.0 ± 15.6	169.6 ± 9.8	170.5 ± 27.8^bd^
Group B	66.9 ± 22.1	71.7 ± 21.4^ac^	87.6 ± 17.4^a^	106.4 ± 21.5^a^	129.1 ± 14.4^a^	165.5 ± 11.6^b^
pFL, deg						
Group A	82.8 ± 10.6	126.8 ± 20.3	150.8 ± 18.9	167.8 ± 9.9	174.0 ± 7.8	175.9 ± 5.5^bd^
Group B	83.2 ± 11.5	90.0 ± 11.3^a^	105.0 ± 14.3^a^	121.4 ± 27.8^a^	140.1 ± 19.7^a^	168.2 ± 9.2^ab^
pER, deg						
Group A	9.0 ± 5.3	25.4 ± 7.1	35.0 ± 8.1	44.0 ± 9.3	51.0 ± 7.1	53.9 ± 6.0^bd^
Group B	10.1 ± 4.6	12.2 ± 4.4^ac^	18.9 ± 5.6^a^	26.0 ± 5.7^a^	35.5 ± 9.2^a^	52.3 ± 7.4^b^
pIR, deg						
Group A	17.7 ± 0.6	15.5 ± 1.1	14.3 ± 1.1	12.0 ± 1.8	9.1 ± 1.0	8.4 ± 0.7^b^
Group B	17.6 ± 0.7	17.5 ± 0.8^ac^	16.0 ± 1.4^a^	14.3 ± 1.2^a^	13.1 ± 1.5^a^	9.5 ± 1.3^ab^
VAS						
Group A	7.7 ± 0.8	3.9 ± 1.1	2.7 ± 1.4	0.9 ± 1.2 ^d^	0.5 ± 0.6	0.1 ± 0.3^b^
Group B	7.7 ± 1.0	4.6 ± 1.6	2.7 ± 1.4	1.2 ± 1.0	0.5 ± 0.7	0.2 ± 0.4^b^
UCLA						
Group A	10.0 ± 1.9			26.8 ± 3.8	32.7 ± 2.0	34.5 ± 0.8^b^
Group B	10.9 ± 1.6			22.3 ± 3.4^a^	26.3 ± 3.6^a^	32.1 ± 2.3^ab^
DASH						
Group A	34.4 ± 2.9			14.2 ± 2.0	9.8 ± 1.5	1.7 ± 1.5^b^
Group B	35.2 ± 2.5			22.5 ± 3.1^a^	17.5 ± 3.5^a^	8.8 ± 2.8^ab^

*pAB* Passive abduction, *pFL* Passive forward flexion, *pER* Passive external rotation, *pIR* Passive internal rotation

^a^ indicate significant difference between group A and group B

^b^ indicate significant difference within the group along with the time points

^c^ indicate no significant difference compared with 0 weeks within the group

^d^ indicate no significant difference compared with 24 weeks within the group

The primary outcome of the study was passive ROM. Passive ROM including abduction, forward flexion, external rotation, and internal rotation within both groups increased over time at specified points after treatment (all *p* < 0.01). At 1 week post-operation, the pAB, pFL, pER, pIR were 123.7 ± 24.9, 126.8 ± 20.3, 25.4 ± 7.1, 15.5 ± 1.1 in group A versus 71.7 ± 21.4, 90.0 ± 11.3, 12.2 ± 4.4, 17.5 ± 0.8 in group B (all *p* < 0.01). At 4 weeks post-operation, there were 144.0 ± 22.8, 150.8 ± 18.9, 35.0 ± 8.1, 14.3 ± 1.1 in group A versus 87.6 ± 17.4, 105.0 ± 14.3, 18.9 ± 5.6, 16.0 ± 1.4 in group B (all *p* < 0.01). At 12 weeks post-operation, there were 159.0 ± 15.6, 167.8 ± 9.9, 44.0 ± 9.3, 12.0 ± 1.8 in group A versus 106.4 ± 21.5, 121.4 ± 27.8, 26.0 ± 5.7, 14.3 ± 1.2 in group B (all *p* < 0.01). At 24 weeks post-operation, there were 169.6 ± 9.8, 174.0 ± 7.8, 51.0 ± 7.1, 9.1 ± 1.0 in group A versus 129.1 ± 14.4, 140.1 ± 19.7, 35.5 ± 9.2, 13.1 ± 1.5 in group B (all *p* < 0.01). At 1 year post-operation, the pFL and pIR were 168.2 ± 9.2, 8.4 ± 0.7 in group A versus 168.2 ± 9.2, 9.5 ± 1.3 in group B (all *p* < 0.01), pAB and pER were similar (all *p* > 0.01) (Table 2). In group A, pAB, pFL, and pER kept increasing from 1 to 24 weeks (all *p* < 0.01). While pIR kept increasing from 1 week to 1 year (all *p* < 0.01). In group B, pAB, pFL, and pIR did not improve at 1 week (*p* > 0.01), then kept increasing along with the time points (all *p* < 0.01). PER kept increasing at the whole followup (all *p* < 0.01) (Table 2).

At all time points, VAS scores of group A were similar with those of group B (all *p* > 0.01) (Table 2), According to previously published manuscript, we selected 1.5 as the MCID of VAS in shoulder [15], the differences of VAS scores between group A and group B at all time points were lower than MCID. and they were lower than pretreatment scores in both groups (all *p* < 0.01). VAS scores were lower than 3 points at Week 4 and then after.

At 12 weeks post-operation, the UCLA scores and DASH scores were 26.8 ± 3.8, 14.2 ± 2.0 in group A versus 22.3 ± 3.4, 22.5 ± 3.1 in group B (all *p* < 0.01). At 24 weeks post-operation, there were 32.7 ± 2.0, 9.8 ± 1.5 in group A versus 26.3 ± 3.6, 17.5 ± 3.5 in group B (all *p* < 0.01). At 1 year post-operation, there were 34.5 ± 0.8, 1.7 ± 1.5 in group A versus 32.1 ± 2.3, 8.8 ± 2.8 in group B (all *p* < 0.01) (Table 2). According to previously published manuscript, we selected 6.0 as the MCID of UCLA scores [15], and 15.91 as MCID of DASH scores [16]. The differences of UCLA scores between group A and group B at 24 weeks was higher than the MCID. These scores significantly improved in both groups at Week 12 and continued to do so until final follow-up (*All p* < 0.01) (Table 2).

## Discussion

There were 2 important findings in the current study. The first was that both corticosteroid hydrodilatation with and without arthroscopic release enhance passive ROM, relieve pain, and effectively improve function in treatment of freezing-phase frozen shoulder. The second was that arthroscopic release with corticosteroid hydrodilatation yield better improvement in passive ROM and function, with similar pain relieve.

Frozen shoulder is marked by acute synovitis and progressive capsular contracture [3, 17]. It is generally acknowledged that inflammation is manifested in early stages of this disease, with collagen and matrix production later culminating in fibrosis [18]. During the early period, synovial hyperplasia and increased vascularity develop, promoting fibrosis of capsular synovium and subsynovial tissue, so-called freezing stage [11]. Compared with control subjects, multiple inflammatory factors are expressed at substantially high levels within joint capsules and subacromial bursae of patients with frozen shoulders [2, 17]. The main aims of the treatment are pain relief and improvement of ROM and function.

Corticosteroid injection is a standard treatment for frozen shoulder proven effective in reducing pain and inflammation by disrrupting inflammatory mediators and synovitis [5, 19]. Unfortunately, this approach is less successful at thawing capsular and rotator interval contractures [20]. In the present study, in group B, pAB, pFL, and pIR did not improve at 1 week, and passive ROM in group A regularly surpassing group B at the former 24 weeks. Single-dose corticosteroid injections are inadequate for long-standing frozen shoulders. A series of three injections at least, and possibly four to six injections, may subsequently be advantageous [1, 21]. In the present study, three injections were performed in both groups. According to Cho and Sun [5, 22], intra-articular space and rotator interval are preferable to subacromial space in this regard, although some authors have achieved similar functional improvement by injecting between intra-articular and subacromial spaces [23]. The inflammation in glenohumeral joint is fundamental for frozen shoulder, subacromial space is also involved [17]. Combined subacromial/intra-articular space injection seems to additively increase the angle of internal rotation [22]. Recent evidence highly recommends hydrodilatation as a choice for treatment of frozen shoulder, contributing greatly to pain relief and functional improvement [4]. Combining hydrodilatation with corticosteroid injection provides superior pain relief in the short term and improvement in ROM across all time frames for frozen shoulder when compared to corticosteroid injection alone [10]. We prefer corticosteroid hydrodilatation, because the high-pressure delivery enhances spread throughout articular cavities, especially along biceps tendon sheath, for broader anti-inflammatory effects.

Arthroscopic capsular release could improve ROM remarkablely, even at Week 1 in group A, the passive ROM significantly improved compared with baseline. PAB, pFL and pER reached 144.0, 150.8, 35.0 in group A at Week 4, and near-normal by postoperative Week 12. Studies have shown that arthroscopic capsular release provides complete and long-lasting improvement in shoulder pain and function, faster than any other therapeutic modality [1, 11–13]. Nevertheless, the extent of capsular release remains in question. Anterior release (including superior glenohumeral ligament and rotator interval) is essential, with some advocating 360° circumferential release [24, 25] and others claiming no demonstrable ROM gains through vaster posterior release [26, 27]. Timing of arthroscopic capsular release is another issue [14, 19, 28]. It is generally agreed that such treatment is indicated for refractory adhesive capsulitis and is appropriate for frozen phase. More recently, however, there is mounting evidence that the timing of intervention does not impact outcomes. Rizvi et al. discovered that unlike patients with protracted symptoms of frozen shoulder, those whose symptoms lasted < 10 months made greater strides in internal rotation after arthroscopic capsular release, with similar outcomes in flexion, abduction, and external rotation, so there was no reason to delay surgery [28]^.^ In another study of 127 patients with frozen shoulders, grouped by symptom duration (onset to surgical intervention), arthroscopic release brought effective and rapid improvement in shoulder motion and function, unrelated to surgical timing [29]. The duration of frozen shoulder was apparently shortened by arthroscopic capsular release [29, 30], reducing the natural disease course by > 12 months [30]. In the present study, passive ROM in group A improved enough to finished most of the daily work at Week 4, and enabling near-normal ROM and shoulder function by postoperative Week 12, the course of frozen shoulder was shortened as a result. That was why we did not continue the follow-up to more than 1 year.

The suitability of arthroscopic capsular release in treating freezing-phase frozen shoulder is still unsettled. Arthroscopic flushing may mitigate inflammatory factors, but cellular infiltrates and hypervascularity are not fully constrained [31]. Consequently, the risk of recurrence is high. This also explains why we had no comparator group expressly for arthroscopic capsular release only. In the present study, arthroscopic release and corticosteroid hydrodilatation complement one another, reaping enhanced effects. Arthroscopic capsular release readily improved ROM, whereas corticosteroid hydrodilatation dampened inflammation in patients of group A, the rapid improvement of ROM was kept. Remarkably, arthroscopic release combined with sequential corticosteroid hydrodilatation had yet to be reported until now.

Although multiple studies have shown that corticosteroid injection in close proximity to arthroscopic surgery raises the risk of infection [32, 33], still other authors using corticosteroid injection in shoulder arthroscopic surgery [10]. In the current study, corticosteroid was mixed with ropivacaine and saline to a relative low concentration. On the other hand, no implants were used in the surgery. Furthermore, arthroscopic shoulder capsule release was not difficult and the surgical time was short. There was no infection in all patients. Anesthesia was imposed on the patient in group A, which may have side effects for them. In the current study, general anesthesia was performed and no side effects was found. Maybe it was because the short time of the surgeries.

There were certain limitations of the present trial, one being the lack of separate comparator groups, one for arthroscopic release only and one for untreated patients. Frozen shoulder is also a progressive disease of spontaneous onset and overlapping phases, so the durations cited by patients may not have been accurate, undermining our results. Then, frozen shoulder developed from injury and diabetes was excluded from the study, which could be a substantial proportion of the frozen shoulder population. And there was potential baseline imbalance in participants with different social engagement, occupation, etc. Likewise, when injecting, leakage of fluid from joint cavities was not totally preventable. Then, the sample size was small, the complications such as infection and cartilage damage were not definite. Then, the follow-up was 1 year, while the natural history of frozen shoulder was longer. Finally, the physician who performed these injections and patients were not blinded to specific protocols, given the obvious group differences in therapeutics.

## Conclusions

Combined arthroscopic release with crticosteroid hydrodilatation would yield better results in passive ROM and function than corticosteroid hydrodilatation only. Future studies should include more participants, and perform long-term follow-up data collection.

## Supplementary Information

**Additional file 1:** .

**Additional file 2:** .

## Data Availability

The datasets supporting the conclusions of this article are included within the article. Raw data can be requested from the corresponding author.

## Abbreviations

ROM: Range of motion
VAS: Visual analog scale
UCLA: University of California, Los Angeles
DASH: Disabilities of the arm, shoulder, and hand
CONSORT: Consolidated standards of reporting trials
SD: Standard deviation
ANOVA: Analysis of variance
