# Supplementary material for: Combined arthroscopic release with corticosteroid hydrodilatation versus corticosteroid hydrodilatation only in treating freezing-phase primary frozen shoulder: a randomized clinical trial
Source: BMC Musculoskelet Disord. 2022 Dec 17;23:1102. doi: 10.1186/s12891-022-06065-3 (PMC9758809; doi:10.1186/s12891-022-06065-3)
Supplement: Supplementary file 1 — Additional file 1: . [file 12891_2022_6065_MOESM1_ESM.docx]

**Original protocol of arthroscopic release with corticosteroid hydrodilatation versus corticosteroid hydrodilatation only in treating freezing-phase primary frozen shoulder**

**Inclusion criteria**

Patients with primary frozen shoulder, aged from 40-70 years old, with normal X-ray findings and global passive motion limitations as follows: <100º forward flexion, <10º external rotation, and internal rotation below L5 level. Freezing-phase frozen shoulder was stipulated as marked nocturnal pain (VAS >7 [scored as 1-10]) of prolonged duration (≥2 months but <9 months), refractory to at least one course of physiotherapy.

**Exclusion criteria**

Shoulder stiffness secondary to previous trauma or surgery; concomitant rotator cuff tear or subacromial impingement proven by MRI; poor general health precluding surgery or corticosteroid injection, and concomitant diabetes.

**Randomization**

An independent physicians performed the recruitment and baseline examination at the out patient department. After baseline examination, the enrollees received one of two sealed opaque envelopes marked as A or B, A would be assigned to combined arthroscopic release/corticosteroid hydrodilatation group (group A), B would be assigned to corticosteroid hydrodilatation only group (group B).

**Sample size calculation**

Sample sizes were determined prior to randomization by test for two means with non-zero null utilizing MedSci sample size tools (version 2.1, Medsci, Shanghai, China). Based on our preliminary study of 15 patients, 36 participants in each group were required to detect a significant difference (2.5 points) in VAS score, with a power of 80% at a type I error level of 0.05, given an expected dropout rate of 20%.

**Group A：combined arthroscopic release and corticosteroid hydrodilatation**

In group A, arthroscopic release and corticosteroid hydrodilatation were performed by the same group of surgeons, rotator interval and anterior glenohumeral joint (including superior, middle, and anterior part of inferior glenohumeral ligaments) was released, then subacromial interval was routinely checked, no debridement and decompression were needed. Full passive ROM was confirmed by manipulation. Injections (as described for group B) were done intraoperatively and at postoperative Weeks 1 and 4.

**Group B：corticosteroid hydrodilatation only**

In group B, triamcinolone acetonide (50 mg) and ropivacaine (100 mg) were mixed in saline to a volume of 20 ml. As an outpatient procedure, we injected 15 ml into glenohumeral joint posteriorly and 5 ml into subacromial space laterally, using anatomic landmarks. Patients were treated in sitting position, marking bodily puncture points and routinely sterilizing the skin. For injecting glenohumeral joint, a 6-cm needle entered 1 cm lateral to and 1 cm below the posterior angle of acromion, 30º relative to coronal plane. Injection was delivered upon sensing a breakthrough, confirmed by intraarticular fluid aspiration and ultrasound. For subacromial injection, a NO 5 needle introduced obliquely (~30º relative to horizontal surface) to the lateral surface of acromion was then moved inferiorly in increments until sensing a breakthrough, then injection was delivered and confirmed by ultrasound. Treatments were repeated 1 and 3 weeks after initial injection.

**Follow-up assessments**

After therapeutic intervention, passive ROM and VAS scores were assessed at Weeks 0 (baseline), 1, 4, 12, 24, and 1 year, obtaining UCLA scores and DASH scores at Weeks 0, 12, 24, and 1 year. Passive ROM including flexion, abduction, external rotation (arm at side), and internal rotation were examined as primary outcome. Passive flexion, passive abduction, and passive external rotation were measured using a goniometer. Passive internal rotation was equated with the highest vertebral level of the back accessible by tip of thumb. A clinical researcher blinded to group allocation was tasked with collection of patient data.
